# Prediction of myopia based on biometric parameters of 500,000 children and adolescents aged 3–18 years

**DOI:** 10.3389/fpubh.2025.1563305

**Published:** 2025-04-16

**Authors:** Siyu Chen, Jingyu Mu, Xiyue Tan, Xiaoxiao Wu, Junguo Duan

**Affiliations:** ^1^Eye School of Chengdu University of Traditional Chinese Medicine, Chengdu, Sichuan, China; ^2^Chengdu University of Traditional Chinese Medicine, Chengdu, Sichuan, China; ^3^Key Laboratory of Sichuan Province Ophthalmopathy Prevention and Cure and Visual Function Protection With Traditional Chinese Medicine Laboratory, Chengdu, Sichuan, China; ^4^Retinal Image Technology and Chronic Vascular Disease Prevention and Control and Collaborative Innovation Center, Chengdu, Sichuan, China

**Keywords:** children, myopia, axial length/corneal radius ratio, cutoff value, axial length

## Abstract

**Purpose:**

To explore the validity and significance of the axial length/corneal radius (AL/CR) Ratio for myopia prediction in children of all ages.

**Methods:**

Between 2020 and 2022, 509,530 children and adolescents aged 3–18 years were sampled in Chengdu City, Sichuan Province, China, by whole-cluster sampling method. Measured their uncorrected visual acuity (UCVA), non-cycloplegic autorefraction, axial length (AL), and corneal radius (CR), Pearson's correlation coefficient and Receiver operating characteristic (ROC) curve were used to determine the accuracy and calculate the cutoff for myopia detection.

**Results:**

The correlation between AL/CR ratio and SE was higher than that between AL and SE at different ages and refractive states. The area under the ROC curve (AUC) of myopia detection by AL/CR ratio (0.9112) was significantly larger than that of AL (0.8923, *P* < 0.001), The difference between boys and girls using AL/CR ratio to detect myopia was also statistically significant (*p* < 0.001). The cutoffs for predicting myopia by AL/CR ratio increased with age, from >2.755 in 3-year-old to >3.095 in 18-year-old, with boys increasing from >2.755 to >3.095, and girls from >2.715 to >3.085, and the cutoff for boys was higher than that for girls at the same age.

**Conclusion:**

Different cutoff for AL/CR ratio can be used to predict myopia for children and adolescents of different ages and genders, and this method can be widely used for clinical diagnosis and mass myopia screening.

## 1 Introduction

Myopia is the most common refractive error condition. An estimated 1.4 billion people suffered from myopia in 2000, and this number is expected to reach 480 million by 2050 (1). China is one of the countries with the highest rates of myopia among children and adolescents (2), and the rate is increasing year by year (3–5) with the growth rate of myopia also accelerating gradually (6). The early onset and high prevalence of myopia jeopardizes the eye health of children and adolescents and may lead to visual impairment and other complications of high myopia (7, 8). Therefore, early screening and monitoring for the prevention and control of myopia remains a public health priority.

Currently, cycloplegic refraction is considered the gold standard for diagnosing myopia in children (9). However, cycloplegic refraction medications may lead to side effects such as allergic conjunctivitis, photophobia, and decreased visual acuity (10–12), Additionally, young children may not cooperate with dilation, parents may be skeptical about it, it is time-consuming and there are instances where ciliary paralysis optometry cannot be successfully completed, making it unsuitable as the preferred method for mass myopia screening. Non-cycloplegic autorefraction and visual acuity examinations are most commonly used in myopia screening, but are less accurate than the gold standard due to factors such as instrumentation and personnel.

The axial length to corneal radius ratio (AL/CR) holds significant advantages over traditional refractive error assessment methods in the screening of myopia in children. AL/CR integrates axial elongation and corneal compensation, addressing the limitations of single biometric parameters, and can effectively identify the risk of myopia. Studies have demonstrated that the correlation between AL/CR and spherical equivalent (SE) is stronger across different age groups compared to axial length (AL) or corneal radius (CR) alone (13, 14). More importantly, AL/CR measurement does not require pupil dilation, is objective and rapid, circumventing errors in refractive examination due to insufficient cooperation from children, making it suitable for large-scale screenings without pharmacological intervention. Additionally, it provides more information such as axial length and corneal curvature radius, which is beneficial for monitoring and preventing fundus diseases. These characteristics make AL/CR an efficient tool for early myopia warning, hyperopia reserve monitoring, and long-term follow-up in the pediatric population (15).

Current studies on AL/CR cutoffs for myopia are limited by small sample sizes (*n* = 300–4,350), narrow age ranges (e.g., 3, 3–4, or 4–6 years), and inconsistent thresholds (2.81–3.08). Standardized cutoffs derived from large, age-stratified populations are needed to improve diagnostic accuracy (13–16). The present study took 3 years to collect ocular biometric data from more than 500,000 individuals in Chengdu, Sichuan Province, aiming to explore the relationship between AL/CR ratio and myopia and the cutoffs in children and adolescents of different ages through large-scale screening, so as to provide reference data for myopia screening and prevention.

## 2 Methods

### 2.1 Participants

In this study, participants were recruited in kindergartens, primary schools, junior high schools, and senior high schools in Chengdu City, Sichuan Province, during the period of 2020–2022 through whole cluster sampling, totalling 509,530. Inclusion criteria comprised: ① kindergarten, primary and secondary school students in Chengdu City, Sichuan Province; ② aged between 3 and 19 years old. Exclusion criteria encompassed: ① patients with various types of glaucoma, corneal disease, lens disease, retinal disease, optic nerve disease, etc.; ② patients with amblyopia, strabismus, significant refractive error, or severe visual dysfunction; ③ blepharophthalmos, severe conjunctivitis, etc.; patients with poor compliance, psychiatric disease, or cognitive impairment.

### 2.2 Ethics statement

This study has been approved by the Ethics Committee of Chengdu University of Traditional Chinese Medicine Ineye Hospital and has obtained informed exemption consent(2019yh-007). All research methods followed the provisions of the Declaration of Helsinki.

### 2.3 Examination procedures

According to the Office of the National Health and Wellness Commission's service specifications for children's eye care and vision screening, eye health examinations for school-age children focus mainly on vision examinations and refractive screening. With the assistance of the Chengdu Education Bureau and the Health Bureau, Chengdu Traditional Chinese Medicine University Ineye Hospital collected information on the students' school type, school name, grade, class, name, gender, age, student status and guardian's phone number. The examination results of each student were recorded through the eye health record system. The members of this study group consisted of ophthalmologists, nurses and optometrists, all of whom received standardized training.

All study subjects underwent an ophthalmological examination, including uncorrected visual acuity (UCVA), non-cycloplegic autorefraction, and cular biometric parameter assessments. Each student underwent an uncorrected visual acuity test using an international standard visual acuity chart for the letter E (GB11533-2011); non-cycloplegic autorefraction was performed using an automated optometrist (modelRM-800; Top-con, Tokyo, Japan), and three measurements were taken in each eye, with a spherical diameter difference of <0.50 D required between any two results, otherwise repeat Measurements were taken and the average of the valid measurements was recorded as the final result; AL Keratometry1 (K1) and Keratometry2 (K2) were analyzed using SUOER Ophthalmic Optical Biometer (SW-9000, Tianjin Shisuowei Electronic Technology Co., Ltd), and three measurements were taken in each eye, which were evaluated by the instrument, and If excessive measurement variability was detected, additional repeated measurements were performed to ensure data accuracy. The average of the three test results was calculated and recorded as the outcome. By averaging K1 and K2 using the formula (K1 + K2)/2, we obtain the Average K. Then, using the formula 337.5/K calculate the CR value. To mitigate potential bias arising from inter-eye correlation, the definition and final analysis included only data from the right eye of each participant.

### 2.4 Definitions

Visual acuity screening and non-cycloplegic autorefraction are simple and quick, widely used for vision screening in children and adolescents, and easy to perform on a wide scale. However, using vision tests alone or non-cycloplegic autorefraction alone is less accurate compared to cycloplegic refraction. Studies have shown that combining both methods to define myopia can achieve optimal accuracy for myopia screening (17, 18). The following definition is therefore used: Myopia was defined as non-cycloplegic SE ≤ -0.50 D+UCVA>0.3 log MAR (age 3), > 0.2 log MAR (ages 4–5), > 0 log MAR (age≥6); the rest of the definitions were non-myopia (19). Myopic refractive states were classified according to SE: low myopia: −3.00 D <SE ≤ −0.50 D, moderate myopia: −6.00 D <SE ≤ −3.00 D, high myopia: SE ≤ −6.00 D (20). Due to the relatively small number of 19-year-olds (804 people), they were categorized in the 18-year-old group.

### 2.5 Statistical analyses

SPSS software (version 27.0) was used for data processing and analysis. Variables following a normal distribution were expressed as mean ± standard deviation, whereas non-normally distributed variables were reported as median with interquartile range (IQR; P25, P75). Comparison of means between groups was performed by *t*-test, and one-way analysis of variance (ANOVA) was used for multiple comparisons. Pearson's correlation coefficient was used to explore the correlation between SE and AL and AL/CR ratios, and the difference was considered statistically significant at *P* < 0.05.

The validity of AL/CR ratio and AL for myopia detection was measured using the Receiver operating characteristic (ROC) curve and calculating the area under the curve (AUC). Calculate the cutoff value, and calculate sensitivity, specificity, and Youden's index.

## 3 Results

### 3.1 General characteristics

Among the 509,530 participants, 263,665 (51.75%) were male and 245,865 (48.25%) were female. The mean age was 10 years, 40.92% were myopic, the mean SE was −0.50 D, the mean AL was 23.68 ± 1.35 mm, the mean CR was 7.82 ± 0.29 mm, and the mean AL/CR ratio was 3.03 ± 0.17. The prevalence of myopia in boys was 39.44%, and that in girls was 42.51%. Moreover, the average central corneal thickness (CCT) is 541.64 ± 33.30 μm, the average anterior chamber depth (ACD) is 3.01 ± 0.34 mm, and the average lens thickness (LT) is 3.63 ± 0.29 mm. The mean SE was lower in girls than in boys, and the AL, CR, and AL/CR ratio were higher in boys than in girls. General characteristics of participants by gender are shown in Table 1.

**Table 1:** General characteristics of participants by gender.

**Variables**	**Total *N* = 509,530**	**Boys *N* = 263,665**	**Girls *N* = 245,865**	***P-*value**
Age (years)	10.18 ± 4.12	10.20 ± 4.10	10.17 ± 4.14	<0.001
LOGMAR vision	0.10 (0.00, 0.52)	0.10(0.00, 0.52)	0.22 (0.00, 0.60)	<0.001
SE (D)	−0.50 (−2.25, 0.25)	−0.38 (−2.25, 0.25)	−0.50 (−2.38, 0.25)	<0.001
AL (mm)	23.68 ± 1.35	23.93 ± 1.33	23.41 ± 1.31	<0.001
K (D)	43.23 ± 1.61	42.89 ± 1.58	43.60 ± 1.57	<0.001
CR(mm)	7.82 ± 0.29	7.88 ± 0.29	7.75 ± 0.28	<0.001
AL/CR	3.03 ± 0.17	3.04 ± 0.17	3.02 ± 0.17	<0.001
CCT (μm)	541.64 ± 33.30	543.45 ± 33.44	539.71 ± 33.05	<0.001
ACD (mm)	3.01 ± 0.34	3.07 ± 0.33	2.95 ± 0.33	<0.001
LT (mm)	3.63 ± 0.29	3.61 ± 0.29	3.65 ± 0.29	<0.001

SE, spherical equivalent; AL, axial length; K, Keratometry; CR, corneal radius; AL/CR ratio, axial length/corneal radius; CCT, Central Corneal Thickness; ACD, Anterior chamber depth; LT, Lens thickness.

In all groups of children and adolescents, the prevalence of myopia increases with age, reaching a maximum at the age of 18 years. From the age of 7–8, the growth rate of myopia began to increase significantly. The growth rate of myopia increased the fastest from 8 to 9 years old, from 24.06 to 36.44%. After that, the growth rate gradually decreased, but it maintained a relatively high growth rate until the age of 14, and then gradually stabilized after the age of 14. Myopia rates for girls are lower than those for boys at ages 3–4 and 6, and higher for girls than for boys at all other ages. The refractive status progressed toward myopia with age, with increasing trends in mean AL and AL/CR ratio, and a relatively stable mean CR with no significant trend. See Table 2.

**Table 2:** Myopia rates, SE, AL, CR, AL/CR ratio by age group.

**Age (years)**	**Myopia rates (%)**			**SE (D)**	**AL (mm)**	**AL/CR**	**CR (mm)**

	**ALL**	**BOYS**	**GIRLS**				
3	6.06	6.20	5.93	0.15 ± 0.82	22.01 ± 0.63	2.83 ± 0.08	7.81 ± 0.31
4	7.38	7.50	7.25	0.19 ± 0.79	22.25 ± 0.65	2.86 ± 0.08	7.79 ± 0.32
5	4.15	4.13	4.18	0.20 ± 0.77	22.49 ± 0.67	2.88 ± 0.08	7.82 ± 0.27
6	7.51	7.52	7.51	0.12 ± 0.86	22.71 ± 0.69	2.91 ± 0.09	7.82 ± 0.29
7	12.77	12.76	12.79	−0.11 ± 1.03	23.02 ± 0.75	2.95 ± 0.10	7.83 ± 0.29
8	24.06	23.32	24.87	−0.46 ± 1.23	23.36 ± 0.83	2.99 ± 0.11	7.86 ± 0.31
9	36.44	34.62	38.45	−0.82 ± 1.42	23.68 ± 0.90	3.03 ± 0.12	7.81 ± 0.30
10	47.67	44.44	51.24	−1.20 ± 1.59	23.94 ± 0.96	3.06 ± 0.12	7.81 ± 0.29
11	56.15	52.76	59.83	−1.57 ± 1.74	24.16 ± 1.01	3.09 ± 0.13	7.83 ± 0.29
12	63.78	60.32	67.51	−1.94 ± 1.90	24.37 ± 1.06	3.12 ± 0.14	7.81 ± 0.30
13	71.69	68.48	75.23	−2.38 ± 2.03	24.59 ± 1.11	3.14 ± 0.14	7.81 ± 0.29
14	76.72	74.50	79.14	−2.77 ± 2.15	24.76 ± 1.16	3.16 ± 0.15	7.81 ± 0.29
15	78.93	76.26	81.72	−2.96 ± 2.21	24.86 ± 1.20	3.17 ± 0.15	7.80 ± 0.29
16	78.44	76.02	81.04	−3.06 ± 2.29	24.90 ± 1.23	3.17 ± 0.17	7.82 ± 0.28
17	80.17	78.27	82.15	−3.30 ± 2.38	25.01 ± 1.26	3.19 ± 0.17	7.82 ± 0.28
18	80.44	77.63	83.51	−3.41 ± 2.45	25.02 ± 1.29	3.20 ± 0.17	7.83 ± 0.33

SE, spherical equivalent; AL, axial length; CR, corneal radius; AL/CR ratio, axial length/corneal radius.

### 3.2 Correlation between AL, AL/CR ratio and SE

**Figure 1:**
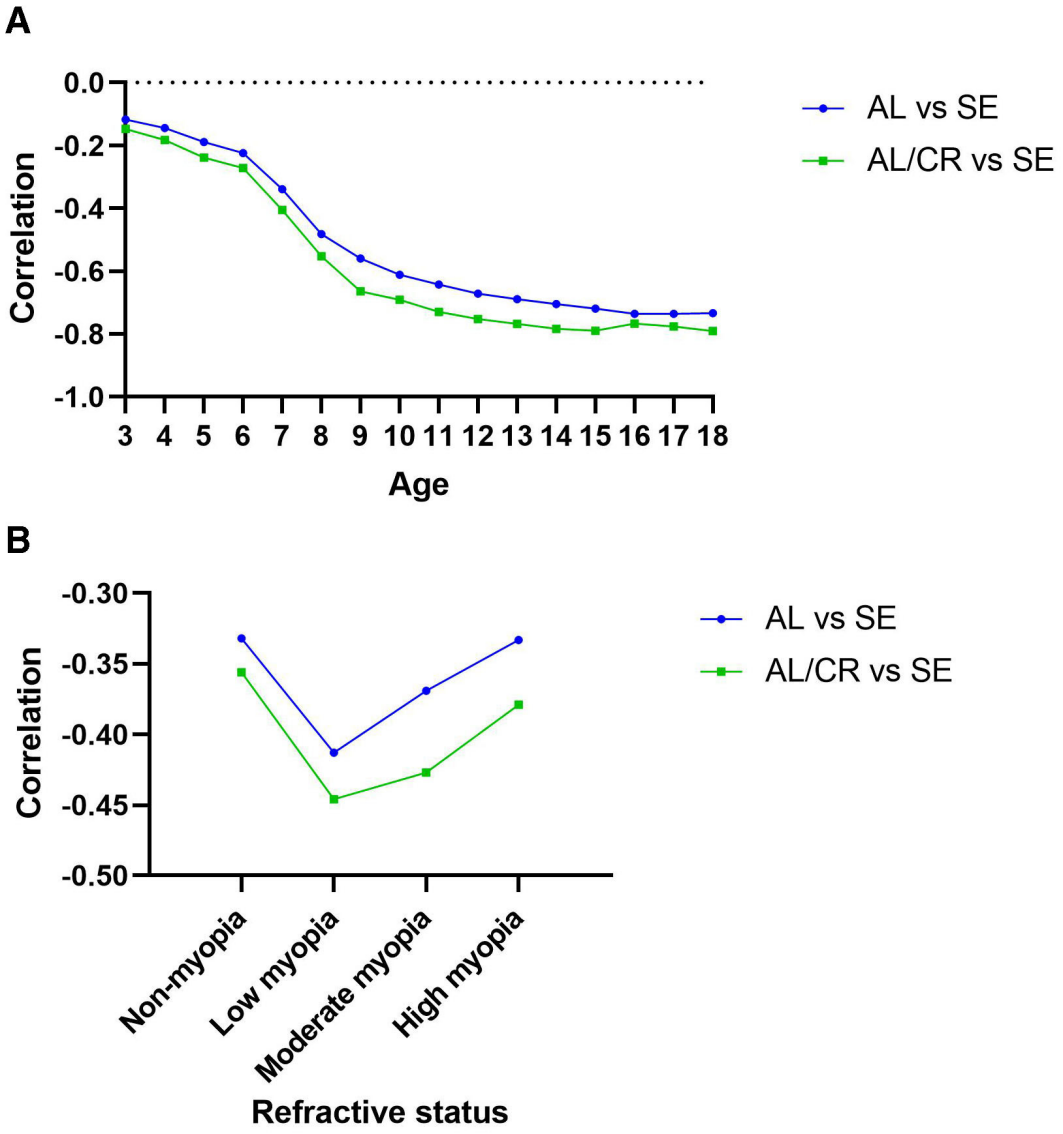
Pearson correlation between SE and AL, AL/CR ratio with 95% CI by age **(A)** or refractive status **(B)**.

The Pearson correlation coefficients between AL, AL/CR ratio, and SE were −0.758 and −0.795, respectively (both *P* < 0.001). The correlation between SE and AL/CR ratio was stronger than the correlation between SE and AL at all ages and all refractive states, see Figure 1. The specific value of Pearson correlation coefficients are shown in the Online Resource (Supplementary Tables S1, S2).

Across age groups, the correlation between SE and AL increased with age from 3 to 18 years (−0.117 to −0.733) and reached a maximum at 17 years (−0.736). The correlation between SE and AL/CR ratio increased with age from 3 to 15 years (−0.147 to −0.789) and then stabilized, reaching a maximum value (−0.790). Under different refractive states, the correlation between SE and AL, as well as the SE and AL/CR ratio, is higher in the myopia group than in the non-myopia group.

### 3.3 ROC curve analysis of myopia detection by age and gender

Table 3 shows the cutoff value, sensitivity, specificity, Youden Index and AUC of AL and AL/CR ratio for myopia detection. Both AL and AL/CR ratio can predict myopia, but the accuracy of using AL/CR ratio is better. The DeLong test comparison shows p <0.001.

**Table 3:** Cutoff value, sensitivity, specificity, Youden Index and AUC for AL and AL/CR ratio for all participants.

**Variables**	**Cutoff**	**Sensitivity**	**Specificity**	**Youden index**	**AUC (95%CI)**	***P*-value**
AL	>23.715	0.740	0.841	0.64	0.8923 (0.8914, 0.8932)	<0.001
AL/CR	>3.045	0.833	0.867	0.70	0.9112 (0.9108, 0.9124)	<0.001

AL, axial length; AL/CR ratio, axial length/corneal radius; 95% CI, 95% confidence interval; AUC, area under the curve.

**Figure 2:**
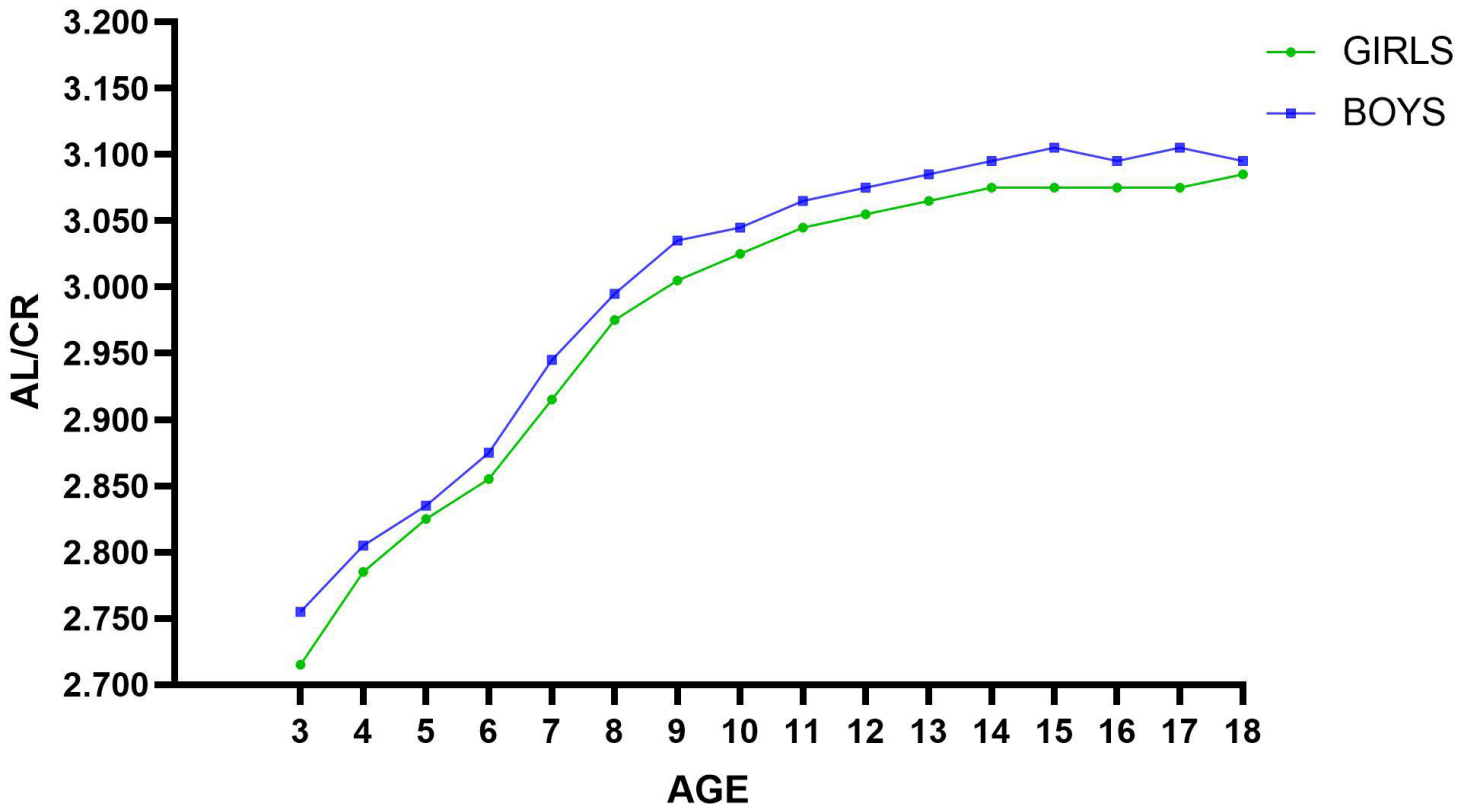
AL/CR cutoff values stratified by age and gender.

The ROC curves for AL/CR ratio and SE were analyzed separately for boys and girls among all participants, with an AUC of 0.911 (0.910 to 0.913, *p* < 0.001) for boys and 0.915 (0.914 to 0.916, *p* < 0.001) for girls, and the difference between boys and girls was significant after the Delong test (Z = −3.901, *p* < 0.001). Therefore, after determining the cutoff value, sensitivity, specificity, Youden index, and AUC value of AL/CR ratio detection by age (see Table 4), calculate them separately for boys and girls according to age (see Tables 5, 6). The AL/CR cutoff values stratified by age and gender are summarized in Figure 2 AL/CR cutoff values stratified by age and gender, with detailed numerical data provided in Online Resource (Supplementary Table S3). The results can provide a reference basis for clinical diagnosis of myopia in children of different gender and ages. The criteria for selecting the cutoffs according to different age groups and gender were as follows: since the sensitivity of AL/CR for predict myopia in all participants was 0.833, this value was adopted as the reference threshold for sensitivity across subgroups. If the sensitivity of the maximum Youden index is ≥0.833, then choose this cutoff; if the sensitivity of the maximum Youden index is <0.833, then choose the best cutoff with sensitivity ≥0.833.

**Table 4:** Cutoff value, sensitivity, specificity, Youden index and AUC of AL/CR ratio for myopia detection by age.

**Age (years)**	**Total (myopia)**	**Cutoff**	**Sensitivity**	**Specificity**	**Youden index**	**AUC (95% CI)**	***P*-value**
3	19,424 (1,178)	>2.755	0.880	0.130	0.010	0.535 (0.518, 0.553)	<0.001
4	35,622 (2,628)	>2.785	0.871	0.142	0.013	0.546 (0.534, 0.558)	<0.001
5	37,147 (1,543)	>2.825	0.855	0.200	0.055	0.613 (0.597, 0.628)	<0.001
6	50,454 (3,791)	>2.865	0.851	0.260	0.111	0.666 (0.656, 0.675)	<0.001
7	41,991 (5,364)	>2.935	0.833	0.488	0.321	0.774 (0.767, 0.782)	<0.001
8	36,556 (8,797)	>2.985	0.851	0.618	0.469	0.825 (0.819, 0.830)	<0.001
9	37,427 (13,637)	>3.015	0.857	0.676	0.533	0.850 (0.846, 0.854)	<0.001
10	34,651 (16,519)	>3.035	0.853	0.699	0.552	0.858 (0.854, 0.862)	<0.001
11	33,296 (18,696)	>3.055	0.840	0.744	0.584	0.869 (0.865, 0.873)	<0.001
12	34,888 (22,253)	>3.065	0.847	0.766	0.613	0.879 (0.876, 0.883)	<0.001
13	32,531 (23,322)	>3.075	0.844	0.772	0.616	0.882 (0.878, 0.886)	<0.001
14	31,031 (23,806)	>3.085	0.843	0.794	0.637	0.891 (0.886, 0.895)	<0.001
15	30,801 (24,311)	>3.085	0.851	0.810	0.661	0.904 (0.900, 0.908)	<0.001
16	24,552 (19,258)	>3.085	0.842	0.809	0.651	0.894 (0.890, 0.899)	<0.001
17	21,037 (16,865)	>3.095	0.835	0.820	0.655	0.894 (0.888, 0.899)	<0.001
18	8,122 (6,533)	>3.095	0.853	0.827	0.680	0.898 (0.890, 0.907)	<0.001

AL/CR ratio, axial length/corneal radius ratio; 95% CI, 95% confidence interval; AUC, area under the curve. Cutoff selection criteria: if the sensitivity of the maximum Youden index is ≥0.833, then choose this cutoff; if the sensitivity of the maximum Youden index is <0.833, then choose the best cutoff with sensitivity ≥0.833.

**Table 5:** Cutoff value, sensitivity, specificity, Youden index and AUC of AL/CR ratio for myopia detection in girls by age.

**Age (years)**	**Total (myopia)**	**Cutoff**	**Sensitivity**	**Specificity**	**Youden index**	**AUC (95% CI)**	***P*-value**
3	9,644 (572)	>2.715	0.956	0.064	0.020	0.546 (0.521, 0.571)	<0.001
4	17,563 (1,273)	>2.785	0.844	0.164	0.008	0.539 (0.522, 0.556)	<0.001
5	18,193 (761)	>2.825	0.844	0.235	0.079	0.613 (0.592, 0.635)	<0.001
6	24,420 (1,833)	>2.855	0.848	0.254	0.102	0.656 (0.642, 0.670)	<0.001
7	20,128 (2,574)	>2.915	0.851	0.425	0.276	0.766 (0.755, 0.777)	<0.001
8	17,542 (4,362)	>2.975	0.851	0.604	0.455	0.823 (0.815, 0.830)	<0.001
9	17,746 (6,824)	>3.005	0.851	0.666	0.517	0.845 (0.839, 0.851)	<0.001
10	16,470 (8,439)	>3.025	0.856	0.699	0.555	0.860 (0.854, 0.866)	<0.001
11	15,961 (9,550)	>3.045	0.841	0.740	0.581	0.868 (0.862, 0.873)	<0.001
12	16,810 (11,348)	>3.055	0.844	0.778	0.622	0.883 (0.878, 0.889)	<0.001
13	15,493 (11,655)	>3.065	0.838	0.780	0.618	0.882 (0.876, 0.888)	<0.001
14	14,830 (11,737)	>3.075	0.841	0.793	0.634	0.888 (0.881, 0.895)	<0.001
15	15,040 (12,291)	>3.075	0.847	0.814	0.661	0.905 (0.899, 0.911)	<0.001
16	11,844 (9,598)	>3.075	0.846	0.822	0.668	0.899 (0.892, 0.906)	<0.001
17	10,306 (8,466)	>3.075	0.846	0.812	0.658	0.901(0.893, 0.908)	<0.001
18	3,876 (3,236)	>3.085	0.850	0.850	0.700	0.906 (0.893, 0.919)	<0.001

AL/CR ratio, axial length/corneal radius ratio; 95% CI, 95% confidence interval; AUC, area under the curve. Cutoff selection criteria: if the sensitivity of the maximum Youden index is ≥0.833, then choose this cutoff; if the sensitivity of the maximum Youden index is <0.833, then choose the best cutoff with sensitivity ≥0.833.

**Table 6:** Cutoff value, sensitivity, specificity, Youden index and AUC of AL/CR ratio for myopia detection in boys by age.

**Age (years)**	**Total (myopia)**	**Cutoff**	**Sensitivity**	**Specificity**	**Youden index**	**AUC (95% CI)**	***P*-value**
3	9,780 (606)	>2.755	0.894	0.110	0.004	0.523 (0.499, 0.547)	=0.058
4	18,059 (1,355)	>2.805	0.837	0.183	0.020	0.552 (0.535, 0.569)	<0.001
5	18,954 (782)	>2.835	0.843	0.201	0.044	0.615 (0.593, 0.637)	<0.001
6	26,034 (1,958)	>2.875	0.854	0.263	0.117	0.677 (0.663, 0.691)	<0.001
7	21,863 (2,790)	>2.945	0.840	0.502	0.342	0.785 (0.774, 0.795)	<0.001
8	19,014 (4,435)	>2.995	0.851	0.630	0.481	0.830 (0.823, 0.837)	<0.001
9	19,681 (6,813)	>3.035	0.841	0.735	0.576	0.860 (0.855, 0.866)	<0.001
10	18,181 (8,080)	>3.045	0.857	0.712	0.569	0.862 (0.857, 0.868)	<0.001
11	17,335 (9,146)	>3.065	0.847	0.755	0.602	0.876 (0.871, 0.882)	<0.001
12	18,078 (10,905)	>3.075	0.852	0.766	0.618	0.884 (0.879, 0.889)	<0.001
13	17,038 (11,667)	>3.085	0.852	0.789	0.641	0.889 (0.884, 0.895)	<0.001
14	16,201 (12,069)	>3.095	0.850	0.807	0.657	0.898 (0.892, 0.903)	<0.001
15	15,761 (12,020)	>3.105	0.840	0.845	0.685	0.910 (0.905, 0.916)	<0.001
16	12,708 (9,660)	>3.095	0.841	0.816	0.657	0.895 (0.889, 0.901)	<0.001
17	10,731 (8,399)	>3.105	0.841	0.816	0.657	0.893 (0.886, 0.900)	<0.001
18	4,247 (3,297)	>3.095	0.879	0.796	0.675	0.898 (0.887, 0.910)	<0.001

AL/CR ratio, axial length/corneal radius ratio; 95% CI, 95% confidence interval; AUC, area under the curve. Cutoff selection criteria: if the sensitivity of the maximum Youden index is ≥0.833, then choose this cutoff; if the sensitivity of the maximum Youden index is <0.833, then choose the best cutoff with sensitivity ≥0.833.

## 4 Discussion

The refractive status of children and adolescents progresses toward myopia with age, with an increasing trend in mean AL and AL/CR ratio and no significant change in CR. Girls had higher myopia and lower mean SE compared to boys, but shorter mean AL and smaller AL/CR ratio. This is consistent with previous studies and the results of a survey conducted in Chengdu City by Wang et al. (17–19). This may be due to the higher lens refractive power of girls (21, 22). The prevalence of myopia increases with age, from 6.06 per cent to 80.44 per cent between the ages of 3 and 18 years, the increase in the rate of myopia has been consistently faster between the ages of 6 and 14 years, especially the fastest between the ages of 7 and 9 years.

Regardless of age and refractive status, the correlation between AL/CR ratio and SE was higher than that between AL and SE. The Pearson correlation coefficients between SE and AL, AL/CR ratio were −0.758 and −0.795, respectively. There were gaps in this correlation coefficient in other studies, For example, in the research of Foo et al. (13), the correlation coefficients of SE and AL, AL/CR ratio are −0.36 and −0.53, in the research of Mu et al. (14), they are −0.667 and −0.754; in the research of Zhao et al. (23), they are −0.82 and −0.90; in the research of He et al. (24), they are −0.657 and −0.881 respectively. This may be due to different sample sizes, geographical differences, and more importantly, different age groups covered by the samples. As can be seen from the present study, the correlations of AL and AL/CR ratio with SE varied in different age groups. Taking the correlation between AL/CR ratio and SE as an example, the correlation changed rapidly in the age range of 3–10 years (−0.147 to −0.691), and the correlation was high and stable in the age range of 10–18 years (−0.729 to −0.790), which may also be attributed to the fact that, in younger children, the lens refraction plays an important role in myopia progression, and with age, lens refraction gradually stabilizes (25). Among the different refractive states, the correlation of myopia was higher than that of the non-myopic group, which is consistent with the findings of He et al. (24). And the correlation was highest in the low myopia group.

The AUC is used to quantify the diagnostic accuracy of biometric parameters in myopia detection, with higher values indicating superior discriminative capacity. In this study, axial length (AL) demonstrated strong predictive performance (AUC = 0.892), while the axial length-to-corneal radius ratio (AL/CR) showed enhanced efficacy (AUC = 0.911). The studies by Mu et al. (14), Wang et al. (17), and other scholars have similarly confirmed that the AL/CR ratio demonstrates significantly superior diagnostic efficacy in myopia assessment compared to single biological parameters (AL) or functional indicators (UCVA), with AUC values reaching 0.937 (vs. AL 0.836) and 0.954 (vs. UCVA 0.928), respectively. This conclusion has been consistently validated in other studies (14, 25). The cutoff of AL used to predict myopia in this study was >23.715, which is very close to the cutoff of 23.71 in the study by Liu et al. (26). The cutoff of AL/CR ratio in this study was>3.045, which is closer to the cutoff of 3.035 in the study by Mu et al. (14). AL/CR ratio >3 was usually considered to be diagnostic of myopia in the past (27–29). However, by calculating the cutoff of AL/CR ratio for myopia assessment at all ages, it was found that myopia could be diagnosed in children aged 3–8 years with an AL/CR ratio of <3. Using AL/CR ratio >3 for all children and adolescents would result in a greater probability of error, especially in underestimation of the degree of myopia in children during the myopia census, and delay in the prevention and treatment of deficient reserve of hyperopia or myopia. From 3 to 18 years of age, the cutoff of AL/CR ratio increases with age, so different cutoff of AL/CR ratio should be used to predict myopia for each age group, and if available by gender. In addition, we found that boys of the same age had higher cutoff than girls regardless of age, which may be due to the fact that boys of the same age had longer AL but lower myopia. This finding is in agreement with Liu et al. (26). There was a difference only at 16 and 18 years of age, in Liu's study the cutoff of AL/CR ratio was higher for girls than boys at 16 years of age and equal at 18 years of age, which may be due to differences in sample sizes and geographic differences.

The AL/CR ratio for detecting myopia in children aged 3–6 years was less judgemental (AUC <0.7), with AUC ranging from 0.545 to 0.656 for girls and from 0.523 to 0.677 for boys between the ages of 3–6 years, with the data for 3 year old boys not being statistically significant. In other studies the data for this age group was also poor compared to other age groups, for example, in Liu's study (26), the AUC for girls were not calculated for ages 3 and 5 due to the small sample size, and were 0.658 (*p* = 0.449) for age 4 and 0.930 for age 6; the data for boys was not statistically significant at the age of 3, and the AUC ranged from 0.729 to 0.937 for ages 4–6. In Liu's study the higher AUC compared to the present study may be due to the fact that Liu used cycloplegic autorefraction as the definition of myopia and the sample size in this age group averaged around 225, which differs from the sample size in the present study. However, it can be consistently concluded that AL/CR ratio alone is not the preferred method of predicting myopia in children aged 3–6 years, probably due to the greater influence of lens thickness on the visual acuity of children in this age group, but if it is not possible to obtain their cycloplegic autorefraction results, the use of AL/CR ratio may still be the best option. For this group of children, it has also been suggested that combining AL/CR ratio with UCVA (14, 17–22) which can be effective in improving the accuracy of predicting myopia. However, in clinical practice, visual acuity examinations may vary due to instrumentation standards, measurement personnel practices, and fluctuations in children's visual acuity, which can be time- and labor-intensive in widespread myopia screening. Therefore, we still recommend that AL/CR be used as the preferred indicator for assessing myopia as much as possible.

The present study was unable to perform cycloplegic autorefraction on all children and adolescents due to the overly large number of eye health screening visits, which may have affected the judgement of refractive status. However, the combination of non-cycloplegic autorefraction and UCVA to jointly define myopia and non-myopia in the present study minimized the error, and the correlation between SE and AL, AL/CR ratio in the overall population is almost consistent with the results of other studies that define myopia using cycloplegic autorefraction, which indicates that the data of the present study were accurate. In addition, the sample size of the present study including 509,530 person-times, makes up for some of the shortcomings of some previous similar studies with relatively small sample sizes and age groups covered. Such as Foo et al. (13) included 349 three-year-old children; Tang et al. (15) included 1,024 children aged 4-6 years; Zhao et al. (16) included 4,350 3- to 4-year-old children in their studies; Mu et al. (14) studied 300 children and adolescents aged 8–18 years; He et al. (24) included 3,922 children aged 6-12 years; and Liu et al. (26) included 7,803 children and adolescents aged 3–18 years. In this study, the cutoff of AL/CR ratio for predicting myopia in each age group of children and adolescents aged 3–18 years were calculated with a large sample size, which provided more accurate data for clinical reference.

In large-scale myopia screening of children and adolescents, cycloplegic autorefraction can be time-consuming, labor-intensive, more costly to communicate, and with uncertain adverse effects. Ocular biometry eliminates the need for ciliary muscle paralysis drops, is unaffected by accommodation, and enables rapid, convenient, and objective acquisition of ocular biometric parameters such as axial length and corneal curvature. Additionally, its lower training requirements for practitioners significantly enhance feasibility for mass screenings in school settings or community programs. This approach also facilitates the monitoring of fundus diseases and promotes public awareness of eye health, contributing to comprehensive ocular health management at a population level. The AL/CR ratio is used for the prediction of myopia with good accuracy, and the cutoff is different for different age groups and genders, so that it can be used as a preferred indicator for myopia prevention and control, census screening, and clinical diagnosis. In future research, we plan to strengthen longitudinal cohort studies to validate the dynamic predictive capacity of the AL/CR ratio during critical developmental stages. Additionally, we encourage further investigations to test AL/CR thresholds across diverse regional populations, thereby improving the generalizability of findings and providing a robust foundation for advancing global myopia management strategies.

## Data Availability

The raw data supporting the conclusions of this article will be made available by the authors, without undue reservation.
