# Supplementary material for: Prediction of myopia based on biometric parameters of 500,000 children and adolescents aged 3–18 years
Source: Front Public Health. 2025 Apr 16;13:1563305. doi: 10.3389/fpubh.2025.1563305 (PMC12040641; doi:10.3389/fpubh.2025.1563305)
Supplement: Supplementary file 1 [file Data_Sheet_1.pdf]

Age(year)	SE vs AL	SE vs AL/CR
3	-0.117	-0.147
4	-0.144	-0.183
5	-0.189	-0.239
6	-0.224	-0.272
7	-0.339	-0.405
8	-0.482	-0.552
9	-0.56	-0.664
10	-0.611	-0.691
11	-0.642	-0.729
12	-0.671	-0.752
13	-0.689	-0.768
14	-0.704	-0.783
15	-0.719	-0.789
16	-0.735	-0.767
17	-0.736	-0.776
18	-0.733	-0.790

Supplementary Table1. Pearson correlation between SE andAL, AL/CR ratio by age

Refractive states	SE vs AL	SE vs AL/CR
Non-myopia	-0.332	-0.356
Low myopia	-0.413	-0.446
Moderate myopia	-0.369	-0.427
High myopia	-0.333	-0.379

Supplementary Table2. Pearson correlation between SE and AL, AL/CR ratio by refractive states

Age(years)	Girls	BOYS
3	2.715	2.755
4	2.785	2.805
5	2.825	2.835
6	2.855	2.875
7	2.915	2.945
8	2.975	2.995
9	3.005	3.035
10	3.025	3.045
11	3.045	3.065
12	3.055	3.075
13	3.065	3.085
14	3.075	3.095
15	3.075	3.105
16	3.075	3.095
17	3.075	3.105
18	3.085	3.095

Supplementary Table3. AL/CR cutoff values stratified by age and gender
